# ERK1/2, MEK1/2 and p38 downstream signalling molecules impaired in CD56^dim^CD16^+^ and CD56^bright^CD16^dim/−^ natural killer cells in Chronic Fatigue Syndrome/Myalgic Encephalomyelitis patients

**DOI:** 10.1186/s12967-016-0859-z

**Published:** 2016-04-21

**Authors:** Teilah Kathryn Huth, Donald Staines, Sonya Marshall-Gradisnik

**Affiliations:** National Centre for Neuroimmunology and Emerging Diseases, Menzies Health Institute Queensland, Griffith University, Southport, QLD Australia; School of Medical Science, Griffith University, Southport, QLD Australia

**Keywords:** Natural killer, Cytotoxic, Cytokine, Intracellular signalling, Mitogen-activated protein kinase signalling, Chronic Fatigue Syndrome/Myalgic Encephalomyelitis

## Abstract

**Background:**

Natural Killer (NK) cell effector functions are dependent on phosphorylation of the mitogen-activated protein kinases (MAPK) pathway to produce an effective immune response for the clearance of target cells infected with viruses, bacteria or malignantly transformed cells. Intracellular signals activating NK cell cytokine production and cytotoxic activity are propagated through protein phosphorylation of MAPKs including MEK1/2, ERK1/2, p38 and JNK. Reduced NK cell cytotoxic activity is consistently reported in Chronic Fatigue Syndrome/Myalgic Encephalomyelitis (CFS/ME) patients and intracellular signalling by MAPK in NK cells remains to be investigated. Therefore, the purpose of this paper was to investigate MAPK downstream signalling molecules in NK cell phenotypes from CFS/ME patients.

**Methods:**

Flow cytometric protocols were used to measure phosphorylation of the MAPK pathway in CD56^bright^CD16^dim/−^ and CD56^dim^CD16^+^ NK cells following stimulation with K562 tumour cells or phorbol-12-myristate-13-acetate plus ionomycin. NK cell cytotoxic activity, degranulation, lytic proteins and cytokine production were also measured as markers for CD56^bright^CD16^dim/−^ and CD56^dim^CD16^+^ NK cell function using flow cytometric protocols.

**Results:**

CFS/ME patients (n = 14) had a significant decrease in ERK1/2 in CD56^dim^CD16^+^ NK cells compared to the non-fatigued controls (n = 11) after incubation with K562 cells. CD56^bright^CD16^dim/−^ NK cells from CFS/ME patients had a significant increase in MEK1/2 and p38 following incubation with K562 cells.

**Conclusions:**

This is the first study to report significant differences in MAPK intracellular signalling molecules in CD56^dim^CD16^+^ and CD56^bright^CD16^dim/−^ NK cells from CFS/ME patients. The current results highlight the importance of intracellular signalling through the MAPK pathway for synergistic effector function of CD56^dim^CD16^+^ and CD56^bright^CD16^dim/−^ NK cells to ensure efficient clearance of target cells. In CFS/ME patients, dysfunctional MAPK signalling may contribute to reduced NK cell cytotoxic activity.

**Electronic supplementary material:**

The online version of this article (doi:10.1186/s12967-016-0859-z) contains supplementary material, which is available to authorized users.

## Background

Natural Killer (NK) cells are innate immune cells which comprise approximately 10–15 % of lymphocytes circulating in the peripheral blood [1]. Two predominant NK cell phenotypes identified by the surface expression of cluster of differentiation (CD) 56 and CD16 and an absence of CD3 provide host immunity through the production of immunoregulatory cytokines and the cytotoxic lysis of target cells [2–4].

Ten percent of peripheral NK cells are CD56^bright^CD16^dim/−^ NK cells which constitutively express receptors for monocyte derived cytokines (monokines) [5, 6]. Monokine receptor ligation rapidly stimulates CD56^bright^CD16^dim/−^ NK cells to produce cytokines including interferon gamma (IFN-γ), tumour necrosis factor alpha and beta (TNF-α and β), granulocyte-macrophage colony-stimulating factor (GM-CSF), interleukin (IL)-10 and IL-13 [5, 6]. CD56^bright^CD16^dim/−^ NK cell cytokine production provides an early source of cytokines which augments NK cell cytotoxic activity and regulates the function of other lymphocytes [2, 4, 5]. Approximately 90 % of peripheral NK cells are cytotoxic CD56^dim^CD16^+^ NK cells [5, 7]. Cytotoxic NK cells contain high numbers of secretory granules which constitutively express apoptotic inducing lytic proteins perforin, granzyme A and granzyme B [3, 8]. Following CD56^dim^CD16^+^ NK cell recognition of a target cell, the lytic proteins are released by a process known as degranulation to induce cytotoxic lysis and subsequent removal of target cells infected with viruses, bacteria or cells which have been malignantly transformed [9, 10].

Unlike T and B lymphocytes, the effector function of NK cells is governed by a myriad of surface receptors which integrate activating or inhibiting signals into intracellular signalling cascades [11–13]. After NK cell receptor ligation, intracellular activation signals are propagated through protein phosphorylation cascades by mitogen-activated protein kinases (MAPKs) [14–16]. Three main subgroups of MAPKs include extracellular signal-regulated kinases (ERK) 1/2, p38 MAPK (p38) and the c-Jun N-terminal kinase (JNK) [14–16]. In response to extra cellular stimuli, the MAPK signalling pathways transduce signals to specific intracellular targets to mediate cellular responses including gene expression, mitosis, motility, cell survival, apoptosis and differentiation [17]. Within the NK cells, phosphorylation of MEK1/2 and p38 regulate cytokine production and ERK1/2 phosphorylation polarises the secretory granule towards the immune synapse for degranulation [16, 18]. In addition to MAPK signalling for normal cellular responses, impairments in MAPK signalling have been suggested to contribute to the pathology of disease processes relating to leukaemia, diabetes, Alzheimer’s and Parkinson’s disease, atherosclerosis, arthritis and airway inflammation [19–25].

Longitudinal reports of significantly reduced NK cell cytotoxic activity in Chronic Fatigue Syndrome/Myalgic Encephalomyelitis (CFS/ME) patients suggests the presence of an NK cell functional deficiency which may contribute to the illness pathogenesis [26–34]. Current investigations into NK cell phenotypes, receptors and lytic proteins in CFS/ME patients have reported equivocal findings and importantly, intracellular signalling by MAPKs in NK cells remains to be examined [27, 35, 36]. Therefore, the purpose of the present study was to investigate NK cell phosphorylation of the MAPK signalling cascade, cytotoxic activity, degranulation, lytic proteins and cytokine production in CD56^dim^CD16^+^ and CD56^bright^CD16^dim/−^ NK cells from CFS/ME patients.

## Methods

### Participant recruitment and inclusion criteria

CFS/ME patients and non-fatigued controls (NFC) were recruited from a participant database at the National Centre for Neuroimmunology and Emerging Diseases, Menzies Health Institute Queensland. All participants completed an online questionnaire based on the 1994 Fukuda definition for fatigue and symptom presentation to determine suitability for study inclusion [37]. From the questionnaire responses, CFS/ME patients meeting the 1994 Fukuda definition and NFC were included. All participants were screened for exclusionary conditions such as epilepsy, thyroid conditions, psychosis, diabetes, cardiac disorders, smoking, pregnant or breastfeeding and immunological, inflammatory or autoimmune diseases. This study was conducted with ethical approval from Griffith University Human Research Ethics Committee (MSC/18/13/HREC) and prior to participation, all subjects provided written informed consent.

### Blood collection and cell isolation

Forty millilitres of sodium heparin blood was collected by venepuncture from the antecubital vein of each participant. To avoid the influence of circadian variation, all blood samples were collected in the morning between 7:30–10 a.m. Laboratory analysis commenced within 4 h of blood collection to maintain cell viability. Routine blood parameters including a full blood count, erythrocyte sedimentation rate, electrolytes and high sensitivity C-reactive protein were assessed on each participant sample by Queensland Pathology. The whole blood samples were diluted with unsupplemented Roswell Park Memorial Institute medium (RPMI) 1640 media (Life Technologies, Carlsbad, USA) and peripheral blood mononuclear cells (PBMCs) were isolated by density gradient centrifugation with Ficoll-Hypaque (GE Health Care, Uppsala, UP).

### NK cell MAPK phosphorylation

Phosphorylation of signalling proteins in the MAPK pathway was examined under two stimulatory conditions using phospho-specific antibodies as previously described [38–40]. Following isolation, the PBMCs in RPMI 1640 media supplemented with 10 % FBS were incubated for a minimum of 2 h at 37 °C with 5 % CO_2_ to reduce background phosphorylation. After resting, the PBMCs were stained with mAbs for CD56-APC (Miltenyi Biotech, Cologne, BG) or CD56-phycoerythrin-cyanine (PE-Cy)7, CD16- brilliant violet (BV)711 and CD3-BV510 (BD Biosciences, San Diego, USA) for 25 min and subsequently washed. The PBMCs were stimulated with either K562 cells (E:T of 25:1) or PMA (50 ng/ml) plus ionomycin (I, 0.5 μg/ml) as a positive control for 15 min in a water bath at 37 °C. A parallel sample of unstimulated (US) cells in RPMI media alone was used to determine basal levels of phosphorylation. BD Phosflow fix buffer 1 (San Diego, USA) containing 4.2 % formaldehyde was pre-warmed to 37 °C and added to the PBMCs, incubated for 10 min at 37 °C and subsequently washed off. The cells were then incubated in BD perm/wash buffer 1 (San Diego, USA) containing FBS and saponin for 10 min which was followed by staining with phosospecific mAbs including signal transducer and activator of transcription (Stat)-3 (pS727)-alexa fluor (AF) 488, MEK1 (pS218)/MEK2 (pS222)-AF488, p38 (pT180/pY182)-PerCP Cy5.5, ERK1/2 (pT202/pY204)-BV421, nuclear factor kappa beta (NF-κβ, pS529)-AF488, inhibitory kappa beta (Iκβ)-AF647, PKCα (pT497)-AF488 and JNK (pT183/pY185)-AF647 for 30 min and subsequent flow cytometry analysis.

### NK cell cytotoxic activity

Flow cytometry was used to measure NK cell cytotoxic activity against the human chronic myelogenous leukaemia K562 cell line as previously described [29, 41]. Briefly, K562 cells (Sigma-Aldrich, St Louis, USA) were cultured in RPMI 1640 media (Life Technologies, Carlsbad, USA) supplemented with 10 % fetal bovine serum (FBS) (Life Technologies, Carlsbad, USA). Following isolation, the PBMCs were stained with Paul Karl Horan-26 fluorescent cell linker dye (Sigma-Aldrich, St Louis, USA) and washed with RPMI supplemented with 10 % FBS. The concentrations of the PBMCs and K562 cells were adjusted to 2.5 × 10^6^ cells/ml and 1 × 10^5^cells/ml respectively and combined at three effector to target (E:T) ratios including 25:1, 12.5:1 and 6.25:1. A control sample of only K562 cells was also included to determine K562 cells undergoing apoptosis not induced by NK cell cytotoxic activity. The PBMCs and K562 cells were incubated for 4 h at 37 °C with 5 % CO_2_ and then stained with fluorescein isothiocyanate (FITC) annexin V and 7-aminoactinomycin (Becton Dickinson [BD] Pharminogen, San Diego, USA) for flow cytometric analysis on a BD Calibur (BD Biosciences, San Diego, USA) dual laser four colour flow cytometer. NK cytotoxic activity was calculated as percent specific death of the K562 cells for the three E:T ratios as previously described [41].

### NK cell degranulation

NK cell surface expression of CD107a and CD107b was measured as a marker for NK cell degranulation as previously reported [9]. PBMCs in the presence of mAbs for CD107a-PE and CD107b-FITC (BD Biosciences, San Diego, USA) were stimulated with either K562 cells (E:T of 25:1) or PMA (50 ng/ml) plus ionomycin (0.5 μg/ml) for 1 h at 37 °C with 5 % CO_2_. Monensin (BD Biosciences, San Diego, USA) was added to the PBMCs and the cells were then incubated for an additional 3 h. An unstimulated control sample included PBMCs incubated in only RPMI 1640 media. Post 4 h incubation, the cells were washed and incubated with mAbs against CD56-APC, CD16-BV711 and CD3-BV510 (BD Biosciences, San Diego, USA) for 25 min which was followed by flow cytometric analysis.

### NK cell lytic proteins and maturation marker

Intracellular staining was used to measure the lytic proteins perforin, granzyme A and granzyme B contained within the secretory granules of NK cells [27, 42]. Surface expression of CD57 was measured as a marker for NK cell maturation [43]. The PBMCs were incubated with mAbs for CD56-PE-Cy7, CD16-BV711, CD3-BV510 and CD57-PE-cyanin-based fluorescent dye (CF)594 for 25 min. The PBMCs were then permeabilised with BD fixation/permeabilisation solution for 20 min, washed in BD perm/wash buffer and then incubated with mAbs including perforin-APC (Miltenyi Biotec, Cologne, BG), granzyme A-FITC and granzyme B-V450 (BD Biosciences, San Diego, USA) for 30 min which was followed by flow cytometric analysis.

### NK cell cytokines

NK cell production of the cytokines IFN-γ, TNF-α and GM-CSF was determined by intracellular staining under two stimulatory conditions as described previously [9, 44]. After isolation, PBMCs were incubated in the presence of either K562 cells (E:T of 25:1) or phorbol-12-myristate-13-acetate (PMA, 50 ng/ml) (Sigma-Aldrich, St Louis, USA) plus ionomycin (I, 0.5 μg/ml) (Sigma-Aldrich, St Louis, USA) for 1 h at 37 °C with 5 % CO_2_. Brefeldin A (BD Biosciences, San Diego, USA) was added to prevent cytokine secretion during stimulation and the cells were incubated for an additional 5 h [9, 44]. PBMCs incubated in RPMI 1640 media alone served as the unstimulated control sample. Following 6 h incubation, the PBMCs were washed and incubated with monoclonal antibodies (mAbs) for CD56-PE-Cy7, CD16-BV711 and CD3-BV510 (BD Biosciences, San Diego, USA) for 25 min. The PBMCs were subsequently washed, incubated in BD fixation/permeabilisation solution (BD Biosciences, San Diego, USA) for 20 min, washed in BD perm/wash buffer (BD Biosciences, San Diego, USA) and then incubated for 30 min with mAbs against IFN-γ- allophycocyanin (APC), TNF-α- peridinin chlorophyll protein-cyanine (PerCP-Cy)-5.5 (BD Biosciences, San Diego, USA) and GM-CSF-PE (Biolegend, San Diego, USA) for flow cytometric detection of intracellular cytokines.

### Multiparametric flow cytometry analysis

Data were collected on a 14-parameter LSR-Fortessa X20 flow cytometer (BD Biosciences, San Diego, USA). Cell signalling technology beads (BD Biosciences, San Diego, USA) were run on a daily basis to ensure optimal flow cytometry performance and application settings were employed to standardise target values for the duration of the experiments. A total of 2500–5000 CD56 positive events were acquired. Data generated for NK cell cytokines, degranulation, lytic proteins and cell maturation was analysed on FlowJo (version 10.0.8) and phosphorylation data were analysed on Cytobank (version 5.0) [45]. NK cell analysis was performed on cells which fell within the lymphocyte population according to forward and side scatter properties. CD56^+^CD3^−^ NK cells were gated to determine total NK cells which was extrapolated to a plot of CD56 and CD16 to identify CD56^bright^CD16^dim/−^ and CD56^dim^CD16^+^ NK cells for the analysis of each marker for cytokines, degranulation, phosphorylation, lytic proteins and cell maturation. A combination of appropriate fluorescence minus one controls, isotype controls matched to antibody concentrations and unstimulated samples were used to determine NK cell gating for each analysis.

### Statistical analysis

Statistical analysis of the data was performed on the Statistical Package for the Social Sciences (version 22) and GraphPad Prism (version 6). All data sets were tested for normality using the Shapiro–Wilk test. The independent Mann–Whitney U test was used to identify any significant differences in the NK cell parameters between the CFS/ME and NFC groups. A Kruskal–Wallis multiple comparisons test was used to identify significant differences in NK cell parameters before and after stimulation within the CFS/ME and NFC cohorts. Significance was set at p < 0.05 and the data is presented as median ± interquartile range unless otherwise stated.

## Results

### Participant inclusion, blood parameters and NK cell phenotypes

14 CFS/ME patients meeting the 1994 Fukuda definition (mean age [years] ± standard error of the mean (SEM) = 53.5 ± 2.17) and 11 NFC (mean age [years] ± SEM = 48.82 ± 3.46) were included in this study. Comparison of the group ages and blood parameters including erythrocyte sedimentation rate, high sensitivity C-reactive protein and full blood counts of white and red blood cells between CFS/ME and the NFC revealed no significant differences (Table 1). Total NK cells were identified as two phenotype populations—CD56^dim^CD16^+^ and CD56^bright^CD16^dim/−^ NK cells—and compared between CFS/ME patients and NFC. No significant differences were observed for NK cell phenotypes (Additional file 1: Figure S1).

**Table 1:** CFS/ME and NFC blood parameters

	CFS/ME (n = 14)	NFC (n = 11)	P value
ESR (mm/h)	7.85 ± 0.77	8.45 ± 1.44	0.700
High sensitivity C-reactive protein (mg/L)	0.99 ± 0.30	0.91 ± 0.41	0.873
White and red blood cells			
White blood cells (10^9^/L)	5.16 ± 0.38	5.26 ± 0.41	0.860
Lymphocytes (10^9^/L)	1.67 ± 0.15	1.67 ± 0.13	1.000
Monocytes (10^9^/L)	0.32 ± 0.03	0.27 ± 0.03	0.258
Neutrophils (10^9^/L)	2.96 ± 0.24	3.15 ± 0.28	0.610
Eosinophils (10^9^/L)	0.17 ± 0.03	0.15 ± 0.03	0.647
Basophils (10^9^/L)	0.03 ± 0.001	0.03 ± 0.001	1.000
Platelets (10^9^/L)	238.54 ± 15.50	248.00 ± 18.01	0.693
Red blood cells (10^12^/L)	4.55 ± 0.12	4.61 ± 0.15	0.755
Haemoglobin (g/L)	138.85 ± 3.82	138.82 ± 4.26	0.996
Haematocrit (%)	0.42 ± 0.01	0.41 ± 0.01	0.493
Mean cell volume (fL)	91.62 ± 0.94	89.27 ± 0.93	0.094
Electrolytes (mmol/L)			
Sodium	137.92 ± 0.46	137.09 ± 0.53	0.249
Potassium	4.10 ± 0.10	4.16 ± 0.12	0.702
Chloride	100.69 ± 0.61	101.64 ± 0.65	0.301
Bicarbonate	28.62 ± 0.63	27.27 ± 0.45	0.112
Anion gap	8.54 ± 0.63	8.36 ± 0.64	0.845

### ERK1/2 significantly reduced in CD56^dim^CD16^+^ NK cells from CFS/ME patients

**Fig. 1:**
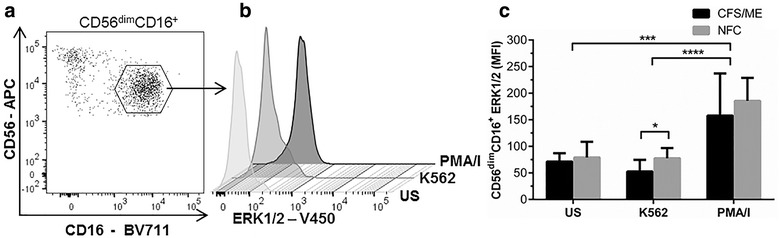
Measurement of the ERK1/2 in CD56^dim^CD16^+^ NK cells. For a representative individual, CD56^dim^CD16^+^ NK cells were identified according to the surface expression of CD56 and CD16 (**a**) which was extrapolated onto a histogram plot to identify median fluorescence intensity (MFI) of ERK1/2 in US, K562 and PMA/I stimulated cells (**b**). Compared to the NFC, phosphorylated ERK1/2 in CD56^dim^CD16^+^ NK cells from CFS/ME patients was significantly reduced (**c**, *p < 0.05). Data are presented as MFI with interquartile range

After incubation with K562 cells at an E:T ratio of 25:1, ERK1/2 was significantly reduced in CD56^dim^CD16^+^ NK cells from CFS/ME patients when compared to NFC (Fig. 1). PMA/I induced a significant increase in ERK1/2 phosphorylation in CD56^dim^CD16^+^ NK cells compared to the US and K562 stimulated cells from CFS/ME and NFC participants. Comparison of ERK1/2 in CD56^bright^CD16^dim/−^ NK cells revealed no significant differences between CFS/ME and NFCs (Additional file 2: Figure S2).

### MEK1/2 and p38 significantly increased CD56^bright^CD16^dim/−^ NK cells from CFS/ME patients

**Fig. 2:**
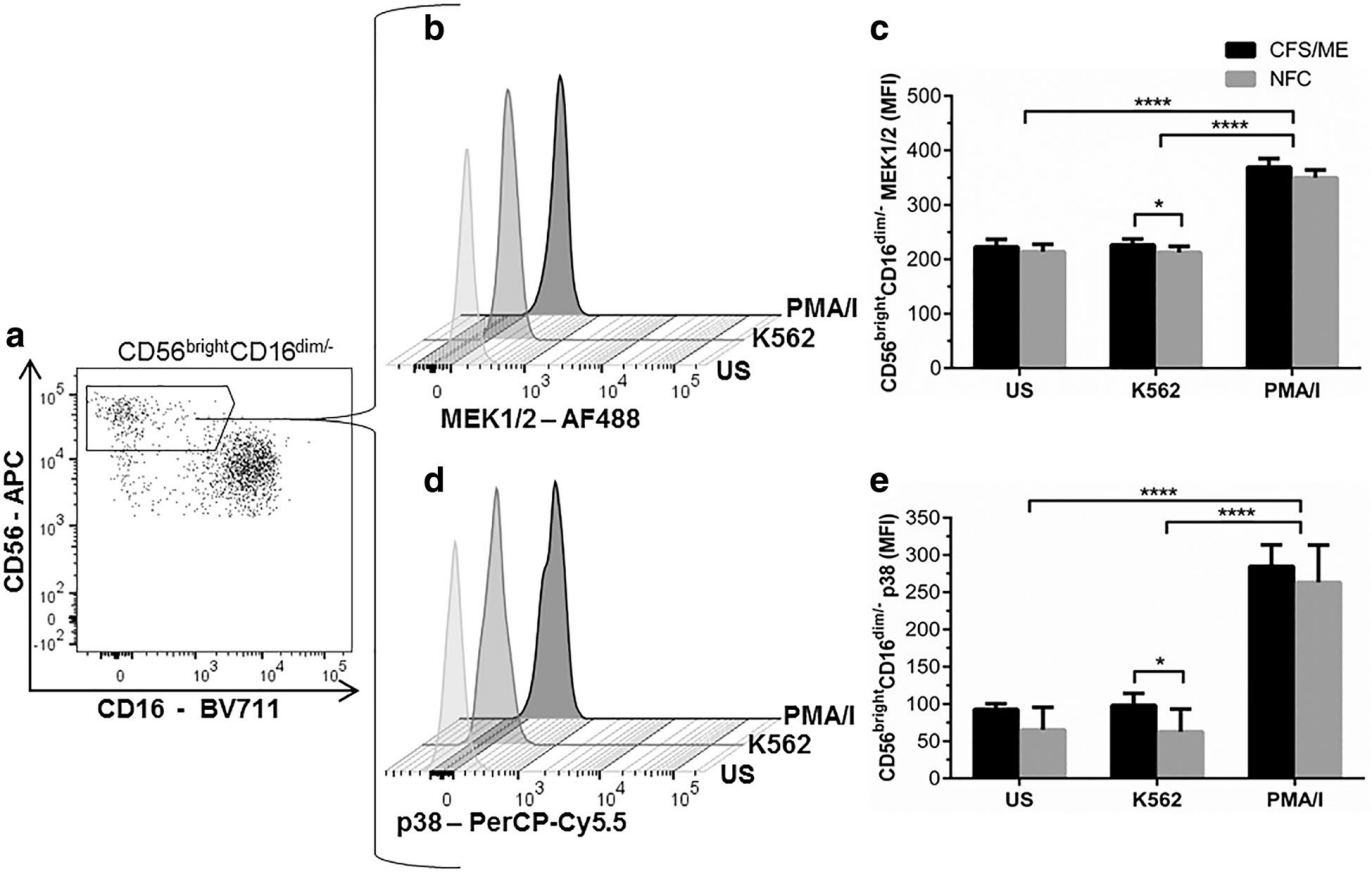
MEK1/2 and p38 measurement in CD56^bright^CD16^dim/−^ NK cells. For a representative individual, CD56^bright^CD16^dim/−^ NK cells were identified using CD56 and CD16 (**a**). CD56^bright^CD16^dim/−^ NK cell MEK1/2 was determined on a histogram (**b**) and comparisons were drawn between CFS/ME and the NFC under different stimulatory conditions (**c**). In comparison to the NFC, MEK1/2 was significantly increased in CFS/ME patients after K562 cell incubation (*p < 0.05). p38 MFI in CD56^bright^CD16^dim/−^ NK cells was also measured on histograms (**d**) and comparisons were drawn against the CFS/ME patients and the NFC (**e**) before and after stimulation. In CFS/ME patients, p38 was significantly increased (*p < 0.05) after K562 incubation compared to the NFC. Data are presented as MFI with interquartile range

In CFS/ME patients, phosphorylation of MEK1/2 and p38 was significantly increased in CD56^bright^CD16^dim/−^ NK cells following incubation with K562 cells at an E:T ratio of 25:1 compared to the NFC (Fig. 2). Stimulation with PMA/I induced a significant increase in MEK1/2 and p38 compared to US and K562 stimulated cells in both CFS/ME and NFC cohorts. Comparison of MEK1/2 and p38 in CD56^dim^CD16^+^ NK cells from CFS/ME and NFC revealed no significant differences (Additional file 2: Figures S3, S4). Measurement of additional MAPK proteins including Stat-3, NF-κβ, Iκβ, protein kinase c-α and JNK revealed no significant differences between CFS/ME and the NFC cohorts (Additional file 2: Figures S5–S9).

### NK cell cytotoxic activity reduced in CFS/ME

In both CFS/ME patients and NFC, NK cell cytotoxic activity at 25:1 was significantly increased compared to 12.5:1 and 6.25:1 ratios. Compared to NFC, CFS/ME was reduced at 25:1 and 12.5 ratios, although this was not statistically significant (Additional file 3: Figure S10).

### CD107a and CD107b increased on CD56^dim^CD16^+^ NK cells after stimulation

Surface expression of CD107a and CD107b on CD56^dim^CD16^+^ and CD56^bright^CD16^dim/−^ NK cells was significantly increased following stimulation with PMA/I and K562 cells in both CFS/ME and NFC (Additional file 3: Figures S11, S12). Comparison of CD107a and CD107b expression between CFS/ME and the NFC under each stimulatory condition revealed no significant differences. CD56^dim^CD16^+^ NK cells from CFS/ME patients displayed increased CD107a following K562 stimulation, although this increase was not significant.

### No significant differences in NK cell lytic proteins from CFS/ME patients

NK cell lytic proteins perforin, granyzme A and granzyme B were measured in CD56^dim^CD16^+^ and CD56^bright^CD16^dim/−^ NK cells from CFS/ME patients and NFC. Comparison between the two groups revealed no significant differences. Surface expression of CD57 was measured as a marker for NK cell maturation on CD56^dim^CD16^+^ and CD56^bright^CD16^dim/−^ NK cells and no significant differences were observed between the CFS/ME patients and the NFC (Additional file 3: Figures S13, S14).

### CD56^dim^CD16^+^ and CD56^bright^CD16^dim/−^ NK cell cytokine production increased after PMA/I stimulation

CD56^dim^CD16^+^ and CD56^bright^CD16^dim/−^ NK cell cytokine production was measured under two stimulatory conditions with PMA/I or K562 cells. INF-γ, TNF-α and GM-CSF production in CD56^dim^CD16^+^ and CD56^bright^CD16^dim/−^ NK cells increased following stimulation with PMA/I in both the NFC and CFS/ME patients (Additional file 4: Figures S15–S17). Comparisons of CD56^dim^CD16^+^ and CD56^bright^CD16^dim/−^ NK cell cytokine production between CFS/ME patients and the NFC under the different stimulatory conditions revealed no significant differences between groups.

## Discussion

This is the first study to investigate ERK1/2 and MEK1/2 MAPK intracellular signalling in CD56^dim^CD16^+^ and CD56^bright^CD16^dim/−^ NK cell phenotypes in CFS/ME. We report novel and significant findings of reduced ERK1/2 in CD56^dim^CD16^+^ NK cells in conjunction with increased MEK1/2 and p38 in CD56^bright^CD16^dim/−^ NK cells. Further investigation of other extracellular signal-regulated kinases will contribute to the understanding of the role of dysregulated MAPK signalling and reduced cytotoxic function of NK cells in CFS/ME. The synergistic functions of both CD56^dim^CD16^+^ and CD56^bright^CD16^dim/−^ NK cells are required for clearance of target cells and dysfunctional signalling through the MAPK pathway in CFS/ME patients may compromise efficient removal of target cells.

CD56^dim^CD16^+^ NK cells from CFS/ME patients had a significant decrease in ERK1/2 which has been identified as an important component for cytotoxic activity due to substrate targeting of paxillin, a cytoskeletal protein kinase [46, 47]. Downstream activation of ERK1/2 is the result of intracellular signalling networks propagating activating signals through phosphorylation cascades [48, 49]. Sequential phosphorylation of MAPK kinase kinase (MAPKKK) and MAPK kinase (MAPKK/MEK1/2) activates ERK1/2 through dual phosphorylation of threonine and tyrosine residues [48, 49]. Phosphorylation of ERK1/2 induces a significant conformational change which is required for NK cell cytotoxic activity as it increases substrate accessibility to phosphorylate paxillin [50, 51]. Paxillin is an adaptor protein which provides a docking site for regulatory proteins such as ERK1/2 and structural proteins including microtubules and actin cytoskeleton [50, 51]. Colocalisation of phosphorylated ERK2 and paxillin to the microtubules and the microtubule organising centre (MTOC) facilitates polarisation of the secretory granules towards the immune synapse [14, 15, 46, 51, 52]. In CFS/ME patients, abnormal signalling through ERK1/2 may interfere with and delay release of the lytic proteins to induce cytotoxic lysis of target cells.

NK cell cytotoxic activity was reduced in the CFS/ME cohort compared to the NFC. The significant reduction of ERK1/2 in CD56^dim^CD16^+^ NK cells may disrupt intracellular signalling required for secretory granule polarisation through the MAPK pathway. As the MAPK cascade integrates signals received from the cell surface, the pathway is subject to complex regulatory and feedback mechanisms which may contribute to the reduction observed in ERK1/2 from CFS/ME patients [46, 53]. ERK1/2 is under constant regulation which also functions to determine specificity of ERK1/2 to target the secretory granules in cytotoxic NK cells [14, 15, 46, 53]. Regulatory mechanisms of ERK1/2 include phosphatases MKP3 and MKPX which dephosphorylate protein tyrosine kinases to inhibit activation [46, 53]. Receptor desensitisation and dissociation of the receptor-ligand interaction changes the strength and duration of activation signals [46, 53]. Scaffold proteins and subcellular localisation of the cascade regulate phosphorylation by directing ERK1/2 to target substrates in the cytoplasm or nucleus [46, 53]. The integration and crosstalk of ERK1/2 with other signalling pathways also acts as a feedback mechanism to regulate phosphorylation levels [46, 53]. As ERK1/2 is subject to a number of distinct mechanisms of regulation, further investigations in CD56^dim^CD16^+^ NK cells from CFS/ME patients are required to determine if these regulatory mechanisms contribute to reduce ERK1/2 phosphorylation.

Degranulation of cytotoxic NK cells was measured to investigate if potential impairments in intracellular signalling through ERK1/2 contribute to reduced cytotoxic activity in CFS/ME patients. Whilst no significant differences were observed in NK cell surface expression of CD107a and CD107b, CD56^dim^CD16^+^ NK cells from CFS/ME patients displayed increased CD107a following K562 stimulation. In support of this current finding, we have previously reported a significant increase in CD107a on NK cells following K562 stimulation in a larger cohort of CFS/ME patients [27]. This finding suggests that the reduction in ERK1/2 may delay movement of the secretory granule and MTOC towards the immune synapse but does not prevent degranulation [14, 15]. Increased degranulation of CD56^dim^CD16^+^ NK cells from CFS/ME patients suggests the cells may be under a continuum of activation due to an inability to induce cytotoxic lysis and subsequent removal of the target cells [27].

Continual activation of NK cells in CFS/ME patients may be the result of prolonged contact with target cells. Kinetic priming facilitated by sustained NK cell contact with target cells retains convergence of the secretory granules and the MTOC at the plasma membrane [54]. This mechanism is known as ‘serial killing’ as subsequent lysis of target cells is more rapid due to pre-docking of the secretory granules, bypassing the need for ERK1/2 to initiate polarisation of the secretory granules towards the immune synapse for degranulation [55]. Further investigations are required to determine if the secretory granule completely fuses with the NK cell membrane to release the entire lytic protein content or if deficiencies in the lytic proteins may contribute to reduced target cell lysis in CFS/ME patients [27, 36, 56]. Reduced perforin and granzyme B has been reported in NK cells from CFS/ME patients which may be a consequence of ‘serial killing’ [27, 36]. Whilst it has been identified that NK cells from CFS/ME patients are degranulating, the inability of NK cells to eliminate target cells by cytotoxic activity suggests that the NK cells may be highly activated through a potential mechanism of inefficient ‘serial killing’.

NK cell production of cytokines including IFN-γ and TNF-α has been identified as an integral part of NK cell cytotoxic activity and increased production of IFN-γ has previously been reported in CFS/ME [27, 29, 57]. NK cells differentiate and mature from CD56^bright^CD16^dim/−^ to CD56^dim^CD16^+^ NK cells with predominant cytokine or cytotoxic effector function [6, 58–60]. This differentiation process suggests that together CD56^bright^CD16^dim/−^ and CD56^dim^CD16^+^ NK cells function to optimise an efficient NK cell response which may be impaired in CFS/ME patients [6, 58–60]. NK cell production of IFN-γ has been reported to augment cytotoxic activity by up-regulating expression of the adhesion molecule ICAM-1 on tumour target cells through the NF-κβ pathway which improves conjugate formation and adherence with cytotoxic NK cells [60]. Conversely, it has also been reported that IFN-γ treatment of tumour cells with high basal levels of ICAM-1, such as K562 cells, up-regulates major histocompatibility class I which acts as a ligand for inhibitory receptors on NK cells and reduces NK cytotoxic activity [60–62]. In CFS/ME patients, further investigations are required to determine if increased IFN-γ may contribute to the proposed inefficient mechanism of ‘serial killing’ resulting in increased degranulation or if IFN-γ desensitises K562 cells to NK cell mediated cytotoxic activity.

Phosphorylation of MEK1/2 and p38 has been implicated in the pathogenesis of many chronic inflammatory diseases and increased production of IFN-γ may be a result of increased MEK1/2 and p38 in CD56^bright^CD16^dim/−^ NK cells from CFS/ME patients [27, 46, 63]. Receptor ligation through environmental stress or innate proinflammatory cytokines including IL-12 and IL-18 initiate MAPK intracellular signalling cascades [17, 64–67]. Similar to ERK1/2 activation, phosphorylation of MEK1/2 and p38 is the result of a tiered protein phosphorylation cascade [17, 64–67]. Activated MEK1/2 in turn phosphorylates ERK1/2, resulting in the formation of ERK2-MEK1 chimera [46, 66, 67]. This chimera is released from its cytoplasmic anchors to undergo a cyto-nuclear shift to initiate IFN-γ production in the nucleus [46, 66, 67]. The phosphorylated ERK2-MEK1 chimera activates c-Fos transcription factor and the activating protein (AP)-1 heterodimer which regulates the IFN-γ gene promoter and subsequent cytokine production [66, 67]. Increased phosphorylation of MEK1/2 may therefore result in increased production of IFN-γ from NK cells in CFS/ME patients as we have previously reported [27, 29, 57]. In contrast to targeting IFN-γ transcription factors, phosphorylated p38 translocates into the nucleus to mediate cytokine production by regulating the half-life of adenylate/uridylate (AU)-rich IFN-γ gene which stabilises and prevents degradation of IFN-γ mRNA [64, 65]. In CD56^bright^CD16^dim/−^ NK cells from CFS/ME patients, an increase in p38 may prolong transcription and translation of IFN-γ [27, 64, 65].

Cytokine synthesis by MEK1/2 and p38 is tightly controlled and each tier of the MAPK signalling cascade is subject to regulation which may be impaired in CFS/ME patients [46, 53]. Phosphatase MKP1 is located in the nucleus and downregulates MEK1/2 and p38 activity by dephosphorylating threonine and tyrosine residues, attenuating cytokine production [46, 53]. Further investigations into the regulation of MEK1/2 and p38 in CD56^bright^CD16^dim/−^ NK cells from CFS/ME patients are required to determine if a regulatory mechanism such as MPK1 may contribute to increased MEK1/2 and p38 activity and IFN-γ cytokine production.

Investigations into the MAPK intracellular signalling pathway in NK cells from CFS/ME patients has revealed novel findings which may explain previous reports of reduced NK cell cytotoxic activity and increased cytokine production. To our knowledge, this is the first study to report significant differences in CD56^dim^CD16^+^ NK cell ERK1/2 from CFS/ME patients. CD56^dim^CD16^+^ NK cell cytotoxic activity is dependent on synergistic action of CD56^bright^CD16^dim/−^ NK cell cytokine production. Consequently, increased MEK1/2 and p38 may increase IFN-γ production which in turn may desensitise K562 cells against NK cell cytotoxic activity in CFS/ME patients. The novel, preliminary findings of this study provide a rationale for further investigations into a larger cohort and particular clinical subgroups of CFS/ME—including severity—to elucidate the cause of reduced NK cell cytotoxic activity.

## Conclusions

The results from this study highlight the importance of intracellular signalling through the MAPK pathway for synergistic function of CD56^dim^CD16^+^ and CD56^bright^CD16^dim/−^ NK cells to ensure efficient clearance of target cells in CFS/ME patients. Further investigations are required to determine if regulatory mechanisms contribute to the aberrations in MAPK intracellular signalling in CD56^dim^CD16^+^ and CD56^bright^CD16^dim/−^ NK cells in a larger cohort of CFS/ME patients.

## Additional files

10.1186/s12967-016-0859-z NK cell phenotype results for CFS/ME patients and NFC.
10.1186/s12967-016-0859-z NK cell MAPK intracellular signalling results for CFS/ME patients and NFC.
10.1186/s12967-016-0859-z NK cell cytotoxic activity, degranulation and lytic protein results for CFS/ME patients and NFC.
10.1186/s12967-016-0859-z NK cell cytokine results for CFS/ME patients and NFC.

## Abbreviations

APC: allophycocyanin
AF: alexa fluor
BD: Becton–Dickinson
BV: brilliant violet
CD: cluster of differentiation
CF: cyanin-based fluorescent dye
CFS/ME: Chronic Fatigue Syndrome/Myalgic Encephalomyelitis
ERK: extracellular signal-regulated kinases
E:T: effector to target
FBS: fetal bovine serum
FITC: fluorescein isothiocyanate
GM-CSF: granulocyte–macrophage colony-stimulating factor
Iκβ: inhibitory kappa beta
I: ionomycin
IFN-γ: interferon gamma
IL: interleukin
JNK: Jun N-terminal kinase
mABs: monoclonal antibodies
MAPK: mitogen-activated protein kinase
MFI: median fluorescence intensity
NK: natural killer
NFC: non-fatigued control
NF-κβ: nuclear factor kappa beta
PBMCs: peripheral blood mononuclear cells
PE: phycoerythrin
PE-Cy: phycoerythrin-cyanine
PerCP-Cy: peridinin chlorophyll protein-cyanine
PMA: phorbol-12-myristate-13-acetate
p38: p38 mitogen-activated protein kinase
RPMI: Roswell Park Memorial Institute
Stat: signal transducer and activator of transcription
TNF: tumour necrosis factor
US: unstimulated
