# Supplementary figures and images for: ERK1/2, MEK1/2 and p38 downstream signalling molecules impaired in CD56dimCD16+ and CD56brightCD16dim/− natural killer cells in Chronic Fatigue Syndrome/Myalgic Encephalomyelitis patients
Source: J Transl Med. 2016 Apr 21;14:97. doi: 10.1186/s12967-016-0859-z (PMC4839077; doi:10.1186/s12967-016-0859-z)

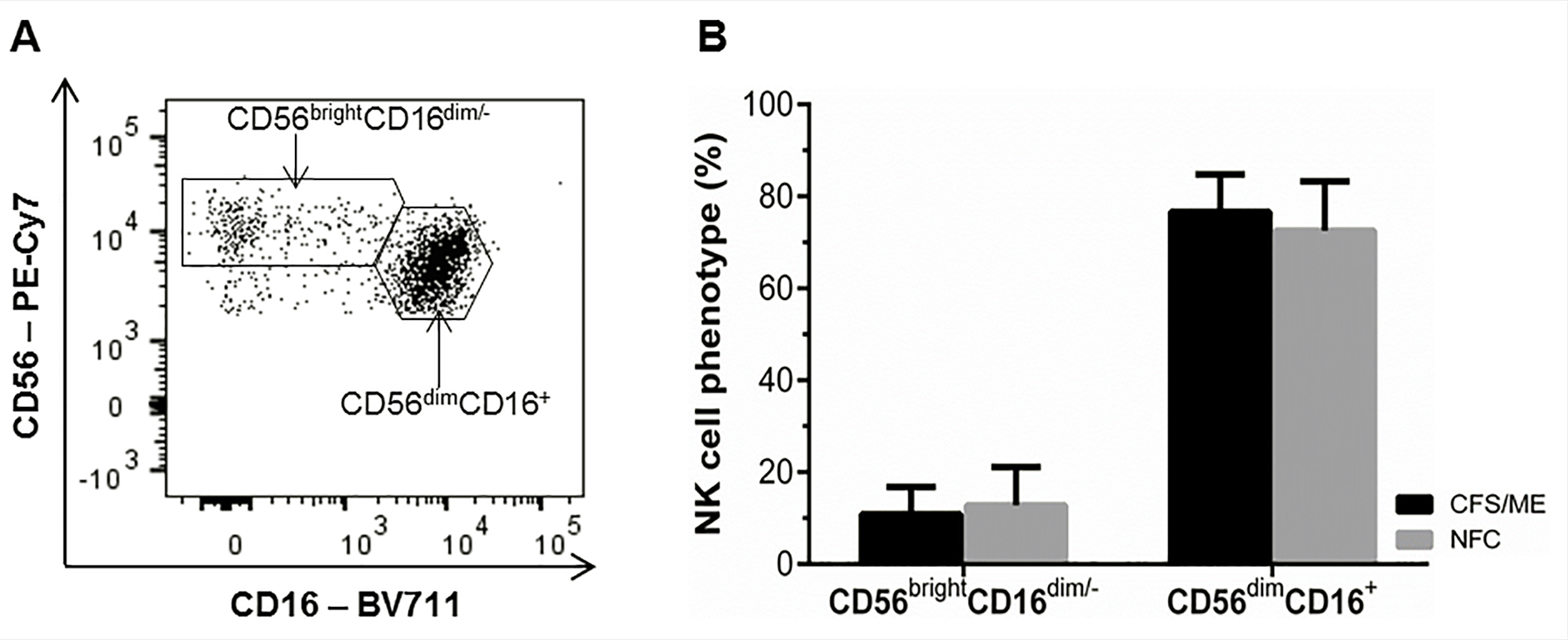

Supplement: Supplementary file 1 — 10.1186/s12967-016-0859-z NK cell phenotype results for CFS/ME patients and NFC. [file 12967_2016_859_MOESM1_ESM.tif]
