# Supplementary material for: ERK1/2, MEK1/2 and p38 downstream signalling molecules impaired in CD56dimCD16+ and CD56brightCD16dim/− natural killer cells in Chronic Fatigue Syndrome/Myalgic Encephalomyelitis patients
Source: J Transl Med. 2016 Apr 21;14:97. doi: 10.1186/s12967-016-0859-z (PMC4839077; doi:10.1186/s12967-016-0859-z)

**Additional File 2**

**Figure S2:** CD56^bright^CD16^dim/-^ NK cell ERK1/2 flow cytometric plot for a representative individual (A). ERK1/2 in CD56^bright^CD16^dim/-^ NK cells were compared between CFS/ME and NFC groups and no significant differences were observed. PMA/I stimulation caused a significant increase in ERK1/2 phosphorylation compared to US (***p<0.001) and K562 incubated cells (****p<0.0001) in both CFS/ME and NFC. Data are presented as MFI with interquartile range.


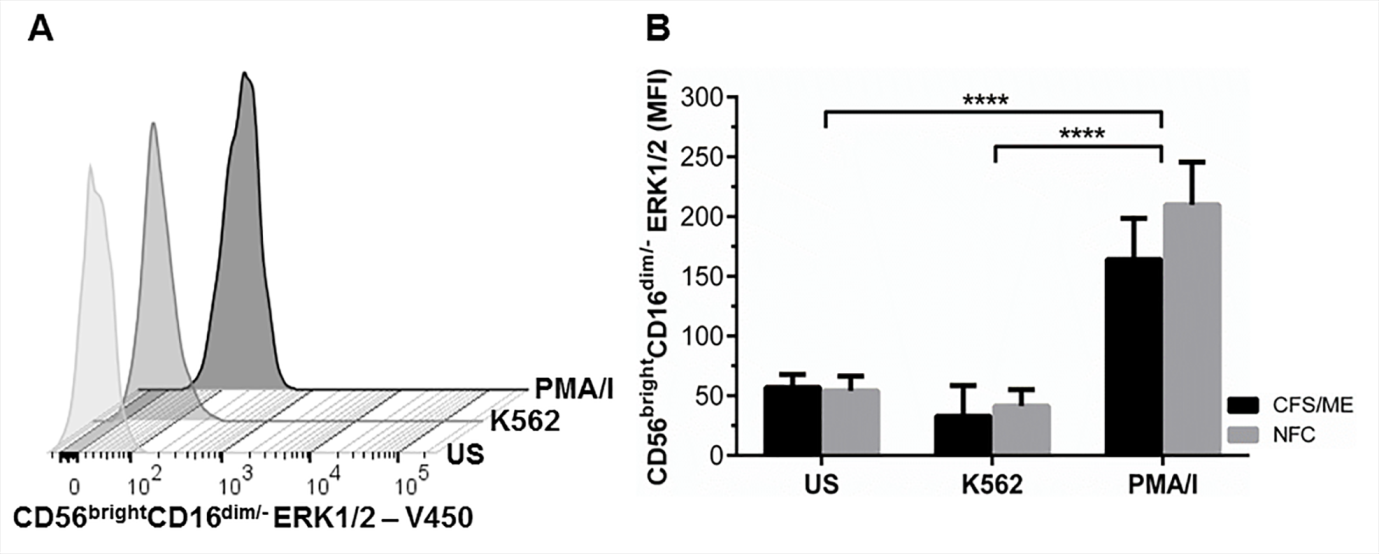


**Figure S3:** Representative flow cytometric plot for MEK1/2 in CD56^dim^CD16^+^ NK cells (A). No significant differences were observed when MEK1/2 were compared between CFS/ME and NFC (B). In both CFS/ME and NFC, PMA/I stimulation resulted in a significant increase in phosphorylated MEK1/2 compared to US (****p<0.0001) and K562 incubation (****p<0.0001). Data are presented as MFI with interquartile range.


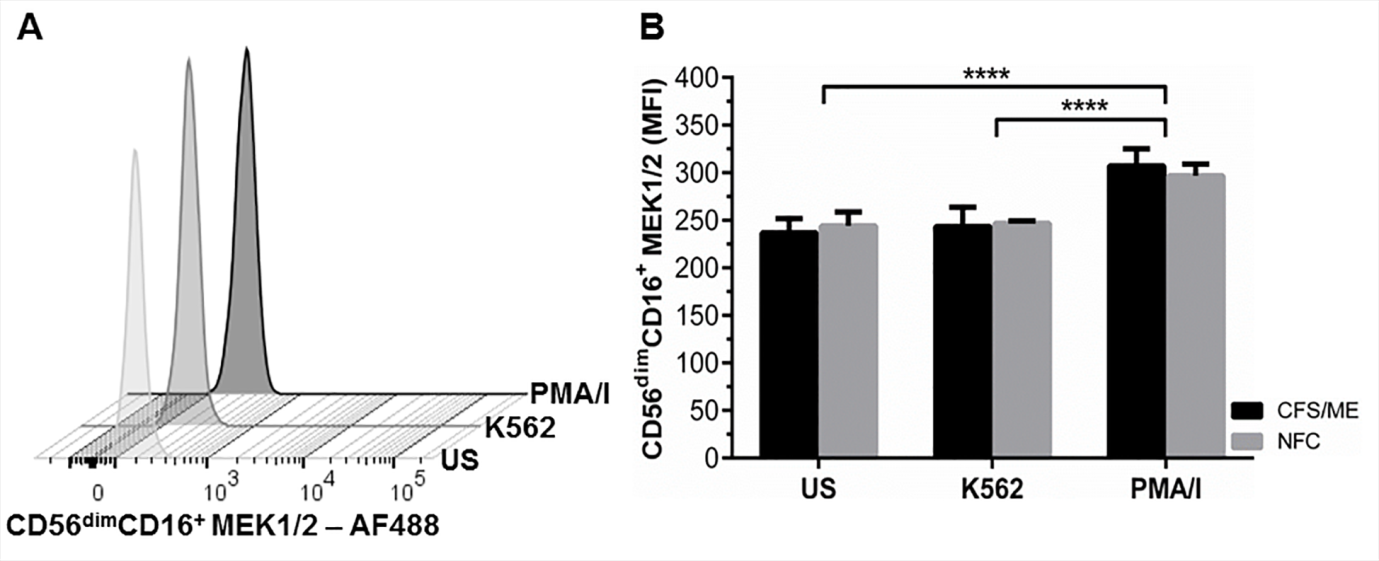


**Figure S4:** p38 representative flow cytometric plot in CD56^dim^CD16^+^ NK cells (A). p38 were compared between CFS/ME and NFC and no significant differences were observed (B). Stimulation with PMA/I caused a significant increase in phosphorylated p38 when compared to US and K562 incubated cells (*p<0.05). Data are presented as MFI with interquartile range.

**
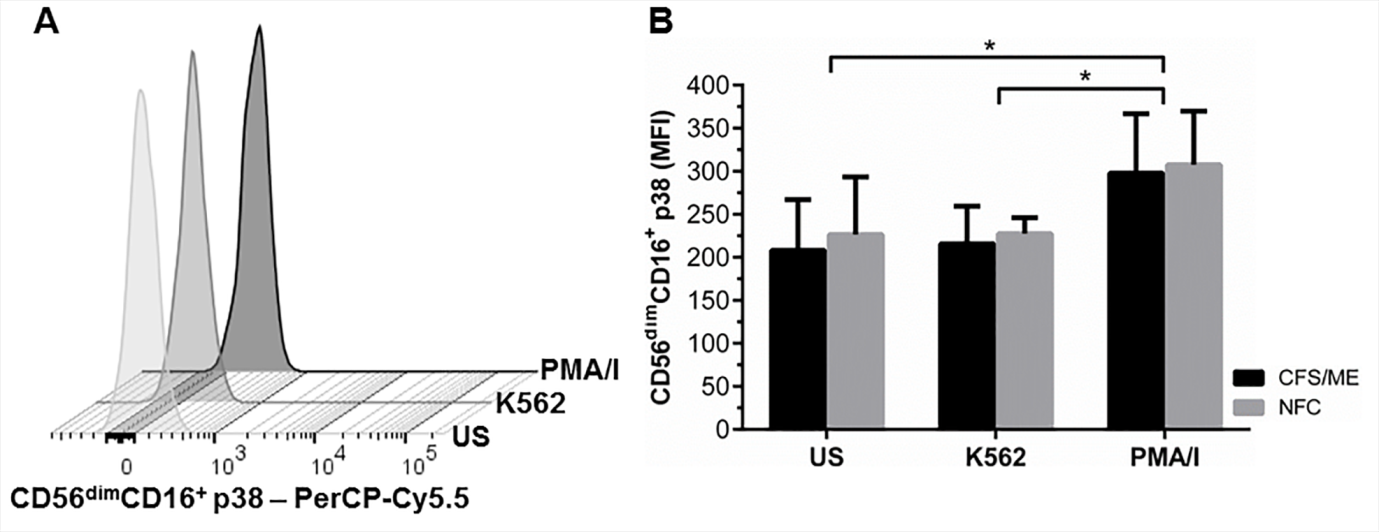
**

**Figure S5:** Representative Stat-3 flow cytometric plots in CD56^dim^CD16^+^ (A) and CD56^bright^CD16^dim/-^ ­(B) NK cells. Comparison of Stat-3 in CD56^dim^CD16^+^ (C) and CD56^bright^CD16^dim/-^ ­(D) NK cells between CFS/ME and NFC revealed no significant differences. In CD56^dim^CD16^+^ and CD56^bright^CD16^dim/-^ ­NK cells, stimulation with PMA/I caused a significant increase in Stat-3 when compared to US (****p<0.0001) and K562 incubated cells (****p<0.0001) in both CFS/ME and NFC.

**
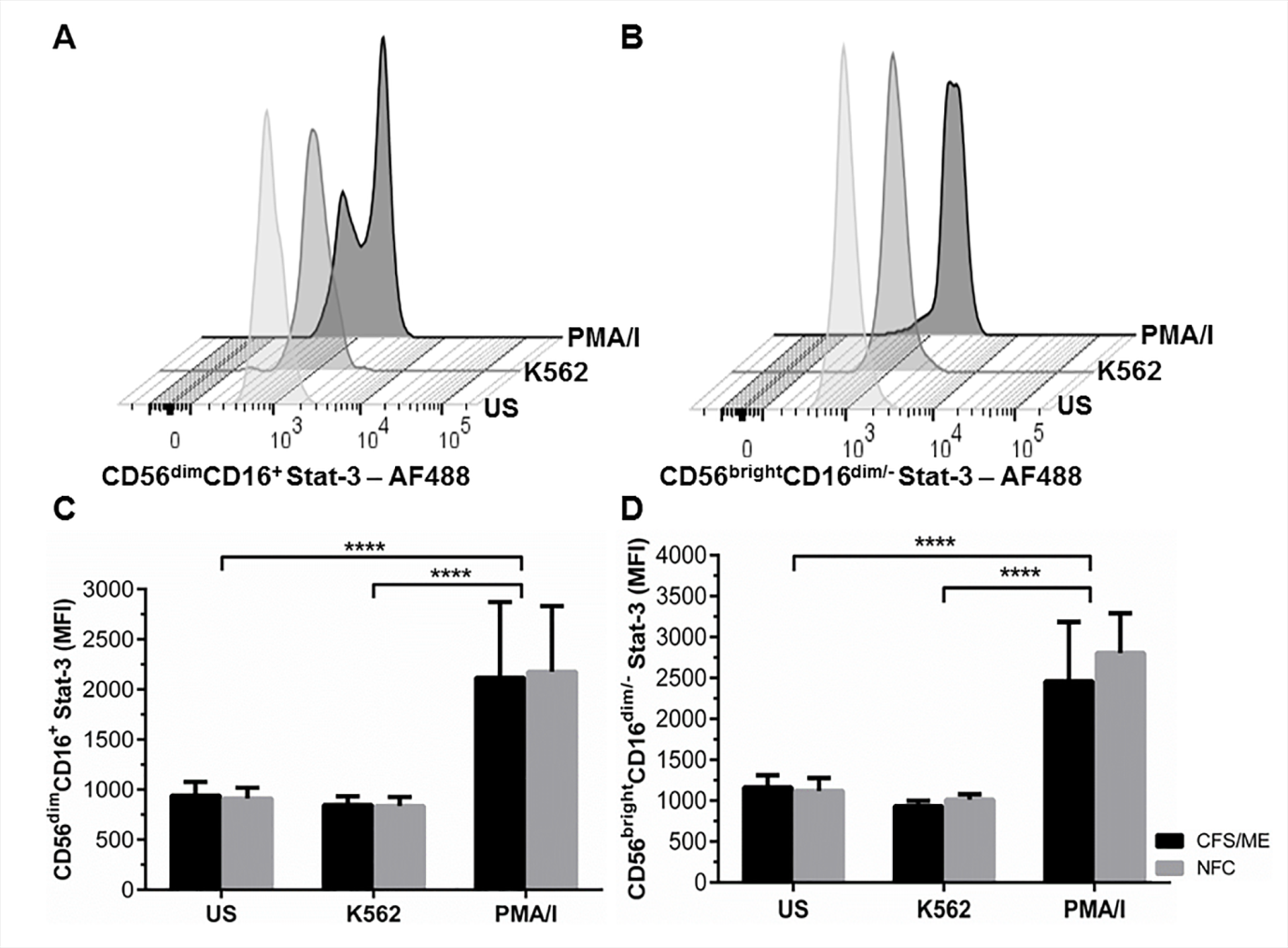
**

**Figure S6:** Representative flow cytometric analysis of NF-κβ in CD56^dim^CD16^+^ (A) and CD56^bright^CD16^dim/-^ ­(B) NK cells. No significant differences were observed when NF-κβ were compared between CFS/ME and NFC in CD56^dim^CD16^+^ (C) and CD56^bright^CD16^dim/-^ ­(D) NK cells. Phosphorylated NF-κβ significantly increased after PMA/I stimulation in both CD56^dim^CD16^+^ (C) and CD56^bright^CD16^dim/-^ ­(D) NK cells compared to US (****p<0.0001) and K562 incubated cells (****p<0.0001) in CFS/ME and NFC. Data are presented as MFI with interquartile range.


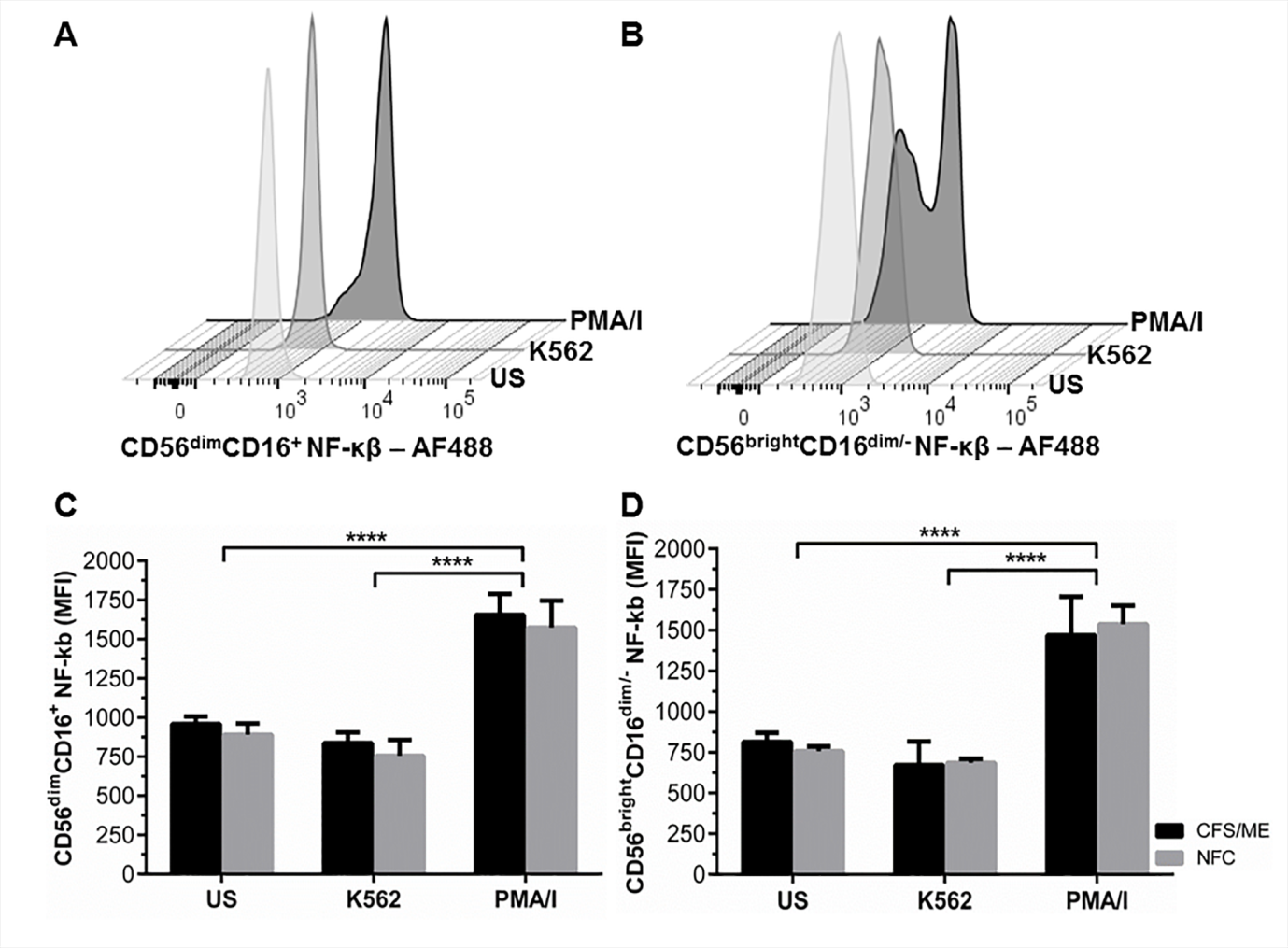


**Figure S7:** Iκβ representative flow cytometric plots in CD56^dim^CD16^+^ (A) and CD56^bright^CD16^dim/-^ (B) NK cells. Iκβ were compared in CD56^dim^CD16^+^ (C) and CD56^bright^CD16^dim/-^ ­(D) NK cells from CFS/ME and NFC and no significant differences were observed. Stimulation with PMA/I caused a significant reduction in Iκβ in both CD56^dim^CD16^+^ (*p<0.05) and CD56^bright^CD16^dim/-^ ­(***p<0.001) NK cells from CFS/ME and NFC. In CD56^bright^CD16^dim/-^ NK cells from CFS/ME patients, incubation with PMA/I also caused a significant reduction (*p<0.05) in Iκβ.


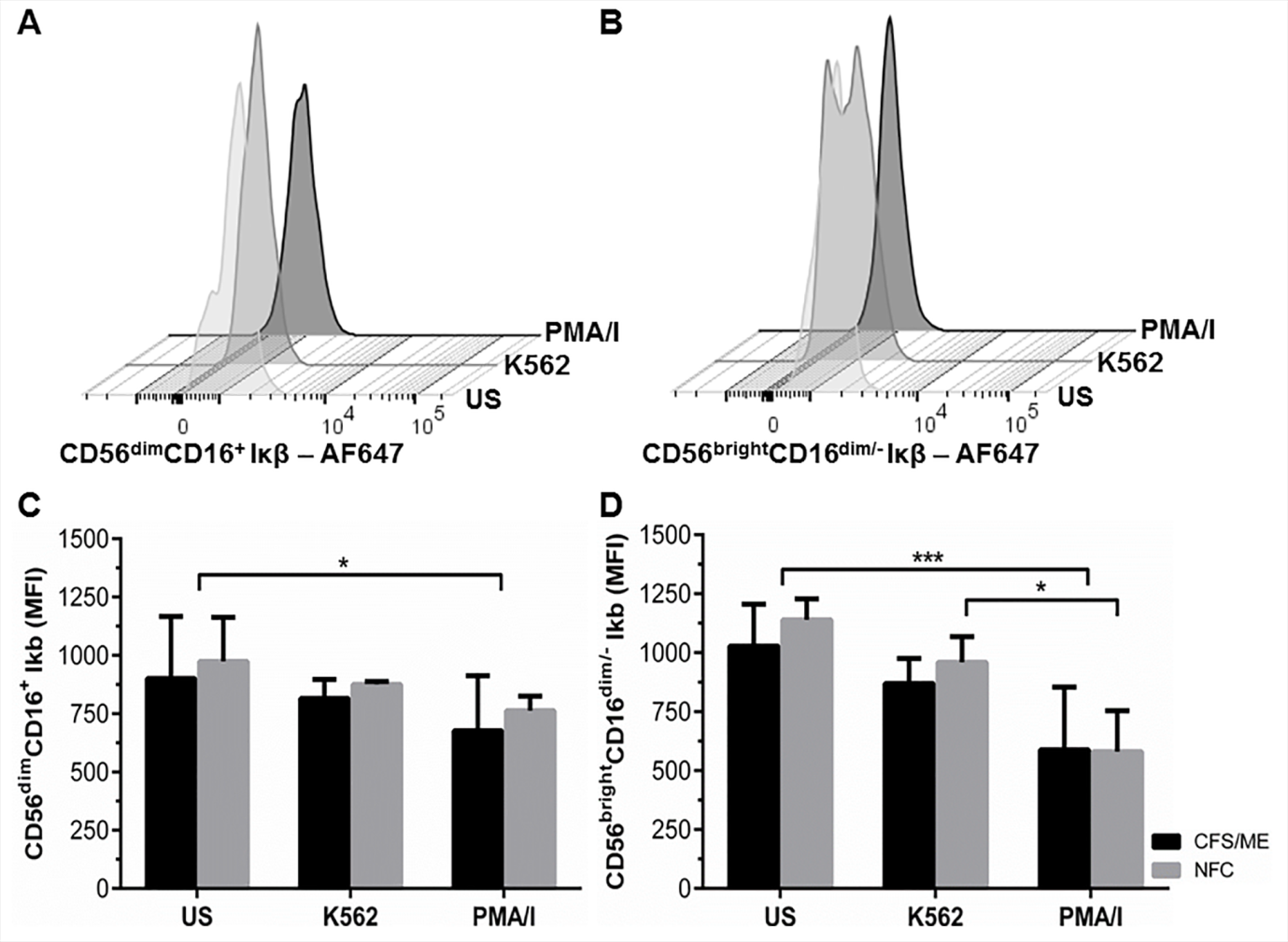


**Figure S8:** Representative flow cytometric plots for the analysis of PKC-α in CD56^dim^CD16^+^ (A) and CD56^bright^CD16^dim/-^ (B) NK cells. PKC-α were compared in CD56^dim^CD16^+^ (C) and CD56^bright^CD16^dim/-^ ­(D) NK cells from CFS/ME and NFC and no significant differences were observed. In CD56^dim^CD16^+^ NK cells from NFC, stimulation with PMA/I caused a significant increase (**p<0.01) in PKC-α phosphorylation compared to K562 cells. PKC-α were significantly increased in CD56^bright^CD16^dim/-^ NK cells after PMA/I stimulation when compared to US (*p<0.05) and K562 ­incubated cells (***p<0.001) in NFC.


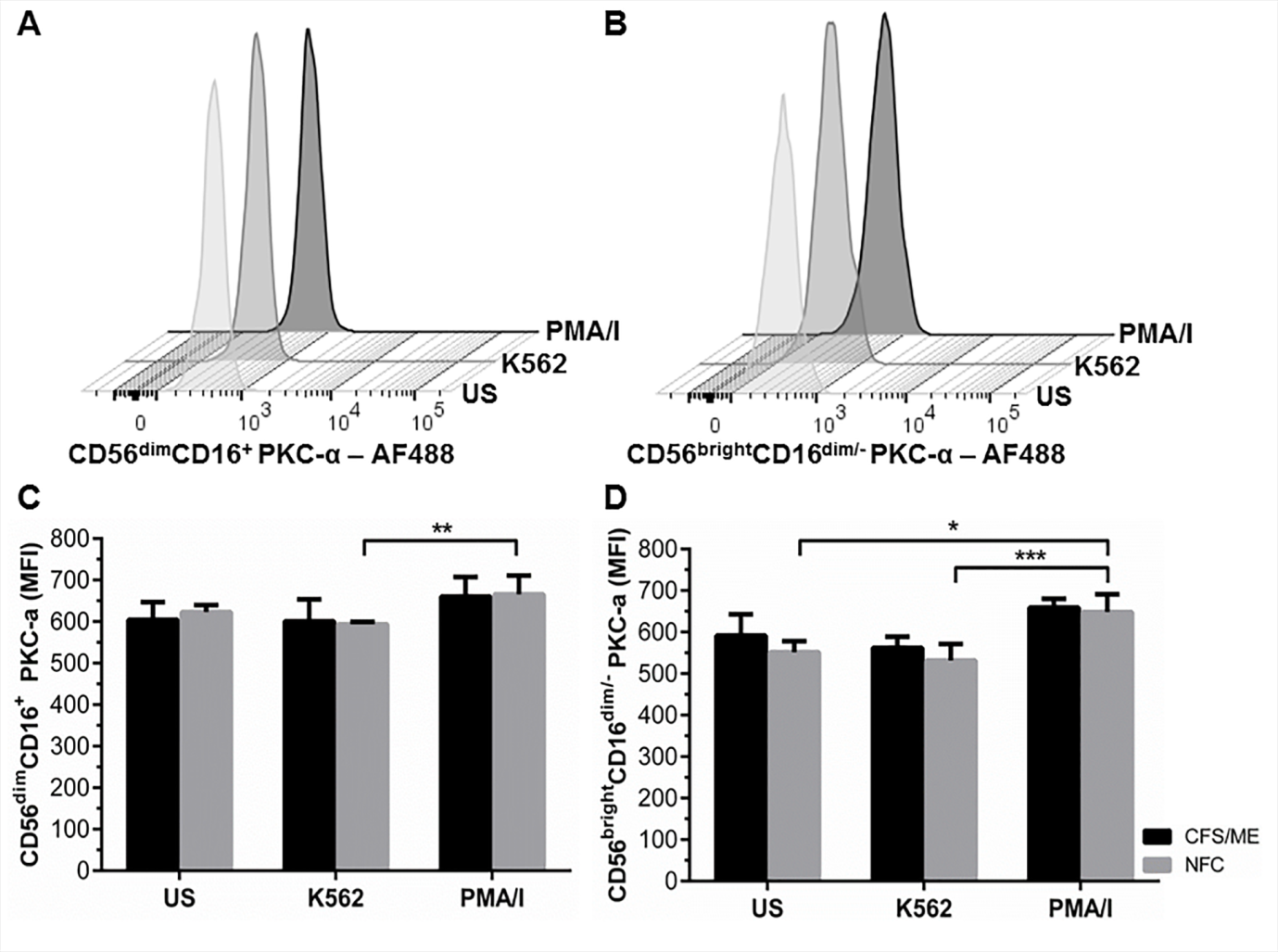


**Figure S9:** Flow cytometric analysis of JNK in CD56^dim^CD16^+^ (A) and CD56^bright^CD16^dim/-^ (B) NK cells. No significant differences were observed when JNK were compared in CD56^dim^CD16^+^ (C) and CD56^bright^CD16^dim/-^ (D) NK cells between CFS/ME and NFC. Significant increases in phosphorylated JNK were observed in both CD56^dim^CD16^+^ (C) and CD56^bright^CD16^dim/-^ (D) NK cells after PMA/I stimulation when compared to US (**p<0.01) and K562 incubated cells ­(***p<0.001) in CFS/ME and NFC.


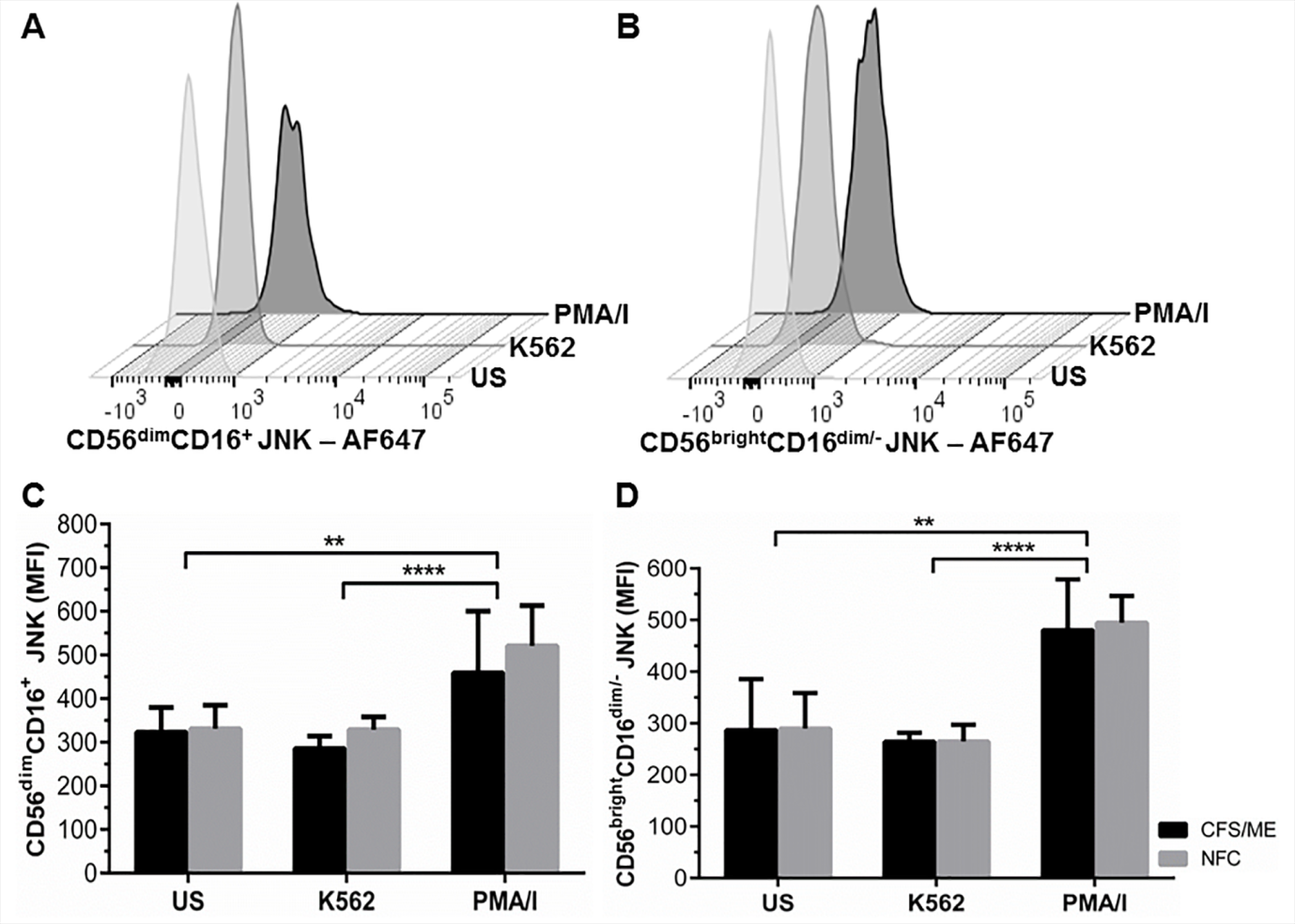

Supplement: Supplementary file 2 — 10.1186/s12967-016-0859-z NK cell MAPK intracellular signalling results for CFS/ME patients and NFC. [file 12967_2016_859_MOESM2_ESM.docx]
