# Supplementary material for: ERK1/2, MEK1/2 and p38 downstream signalling molecules impaired in CD56dimCD16+ and CD56brightCD16dim/− natural killer cells in Chronic Fatigue Syndrome/Myalgic Encephalomyelitis patients
Source: J Transl Med. 2016 Apr 21;14:97. doi: 10.1186/s12967-016-0859-z (PMC4839077; doi:10.1186/s12967-016-0859-z)
Supplement: Supplementary file 3 — 10.1186/s12967-016-0859-z NK cell cytotoxic activity, degranulation and lytic protein results for CFS/ME patients and NFC. [file 12967_2016_859_MOESM3_ESM.docx]

**Additional File 3**

**Figure S10:** NK cell cytotoxic activity in CFS/ME and NFC at three E:T ratios. In both CFS/ME patients and NFC, NK cell cytotoxic activity at 25:1 was significantly increased compared to 12.5:1 (*p<0.05) and 6.25:1 ratios (**p<0.01).

**
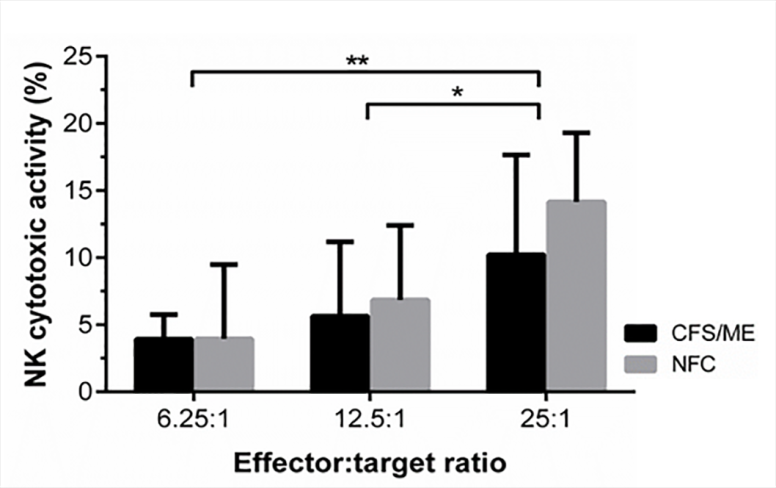
**

**Figure S11:** Representative flow cytometry plots for CD107a in CD56^dim^CD16^+^ (A) and CD56^bright^CD16^dim/-^ (B) NK cells. CD107a were measured in US cells and after stimulation with either K562 cells or PMA/I. Comparison of CD107a on CD56^dim^CD16^+^ (C) and CD56^bright^CD16^dim/-^ (D) NK cells between CFS/ME and NFC revealed no significant differences. CD107a expression significantly increased after K562 and PMA/I (****p<0.0001) stimulation in CD56^dim^CD16^+^ NK cells from both CFS/ME and NFC cohorts. In CD56^bright^CD16^dim/-^ NK cells, PMA/I stimulation significantly increased expression of CD107a when compared to K562 and US cells (****p<0.0001) from CFS/ME and NFC cohorts.


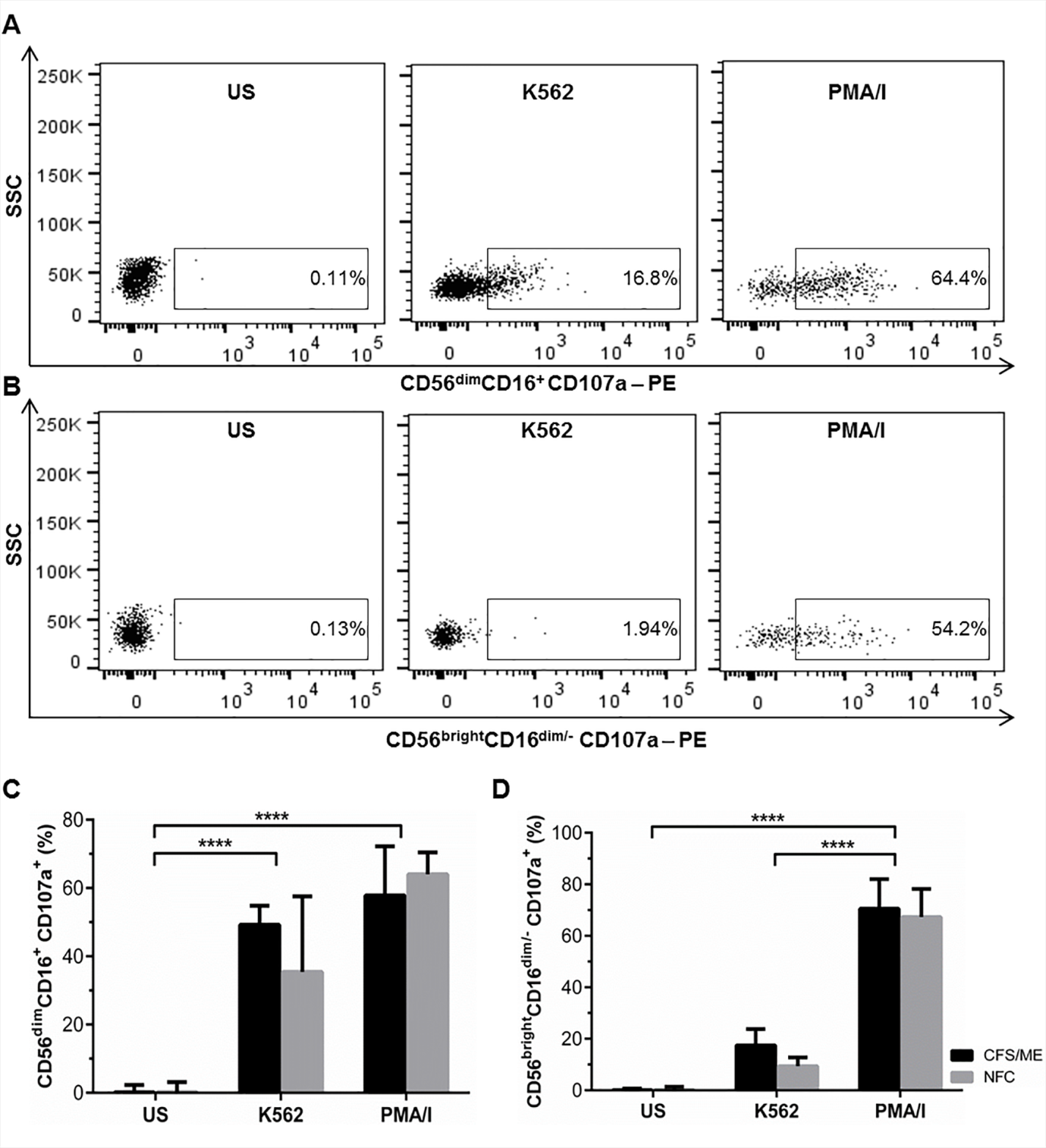


**Figure S12:** Flow cytometric analysis of CD107b on CD56^dim^CD16^+^ (A) and CD56^bright^CD16^dim/-^ (B) NK cells. No significant differences were observed when CD107b expression were compared between CFS/ME and NFC on CD56^dim^CD16^+^ (C) and CD56^bright^CD16^dim/-^ (D) NK cells. In CD56^dim^CD16^+^ NK cells, stimulation with K562 cells (*p<0.05) and PMA/I (****p<0.0001) caused a significant increase in CD107b expression in both CFS/ME and NFC compared to US. PMA/I stimulation significantly increased CD107b expression on CD56^bright^CD16^dim/-^ NK cells from CFS/ME and NFC when compared to K562 and US (****p<0.0001).


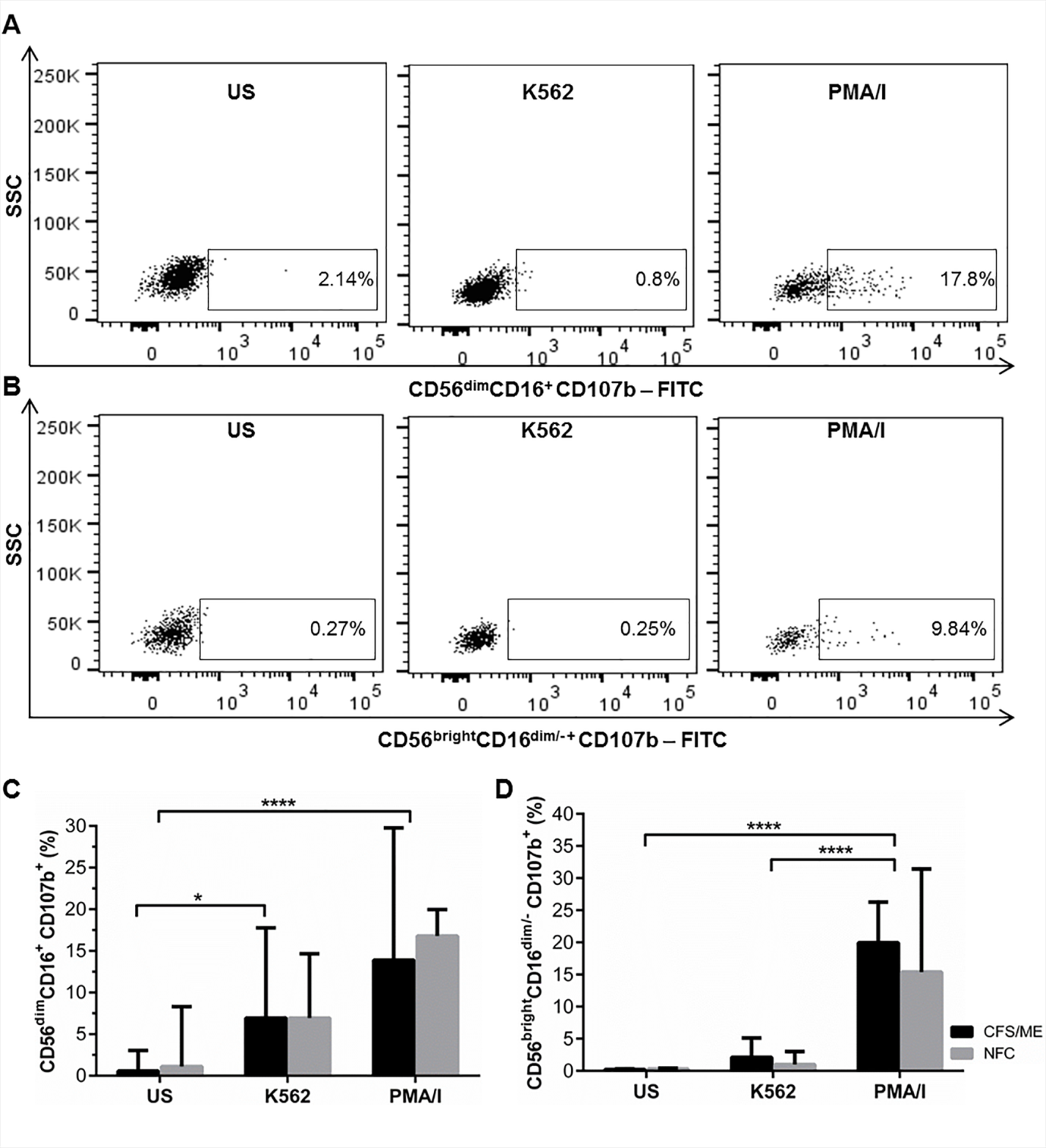


**Figure S13:** Flow cytometric example of CD56^dim^CD16^+^ NK cell perforin (A), granzyme A (B), granzyme B (C) and CD57 (D). Comparison of the lytic proteins and CD57 between CFS/ME and NFC revealed no significant differences (E).

**Figure S14:** Representative flow cytometric plots for CD56^bright^CD16^dim/-^ NK cell expression of perforin (A), granzyme A (B), granzyme B (C) and CD57 (D). No significant differences were observed when the lytic proteins and CD57 expression were compared between CFS/ME and NFC (E).
