# Supplementary material for: ERK1/2, MEK1/2 and p38 downstream signalling molecules impaired in CD56dimCD16+ and CD56brightCD16dim/− natural killer cells in Chronic Fatigue Syndrome/Myalgic Encephalomyelitis patients
Source: J Transl Med. 2016 Apr 21;14:97. doi: 10.1186/s12967-016-0859-z (PMC4839077; doi:10.1186/s12967-016-0859-z)

**Additional File 4**

**Figure S15:** Representative flow cytometric plots for CD56^dim^CD16^+^ (A) and CD56^bright^CD16^dim/-^ (B) NK cell production of IFN-γ. Comparison of IFN-γ production in CD56^dim^CD16^+^ (C) and CD56^bright^CD16^dim/-^ (D) NK cells between CFS/ME and NFC revealed no significant differences. IFN-γ production significantly increased after PMA/I stimulation in both CD56^dim^CD16^+^ and CD56^bright^CD16^dim/-^ NK cells when compared to US and K562 incubated cells (****p<0.0001).


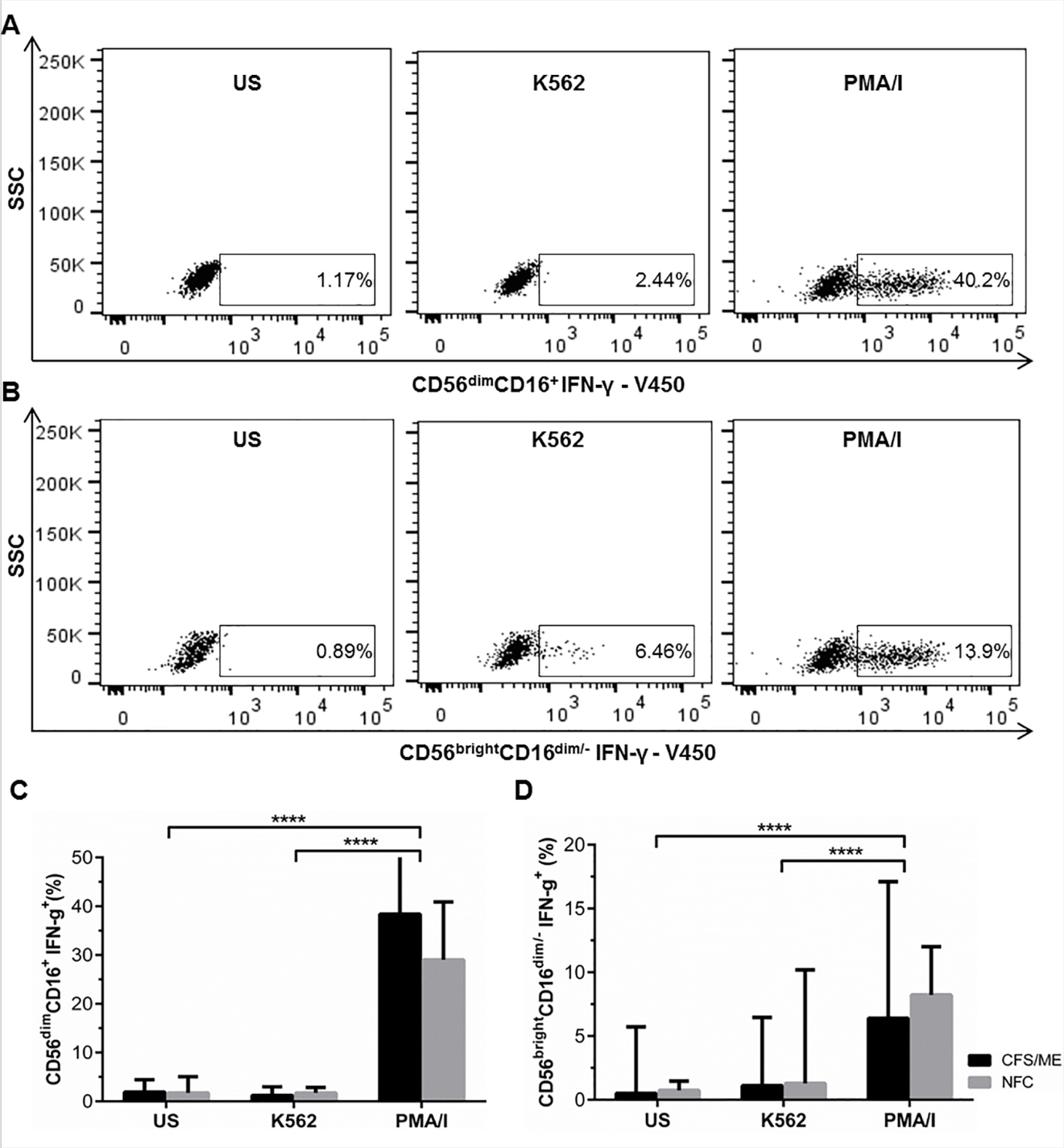


**Figure S16:** Flow cytometric plots for TNF-α in CD56^dim^CD16^+^ (A) and CD56^bright^CD16^dim/-^ (B) NK cells. Between CFS/ME and NFC cohorts, TNF-α production in CD56^dim^CD16^+^ (C) and CD56^bright^CD16^dim/-^ (D) NK cells were not significantly different. In CD56^dim^CD16^+^ NK cells, PMA/I stimulation significantly increased TNF-α production when compared to US and K562 incubated cells (****p<0.0001) in both CFS/ME and NFC.


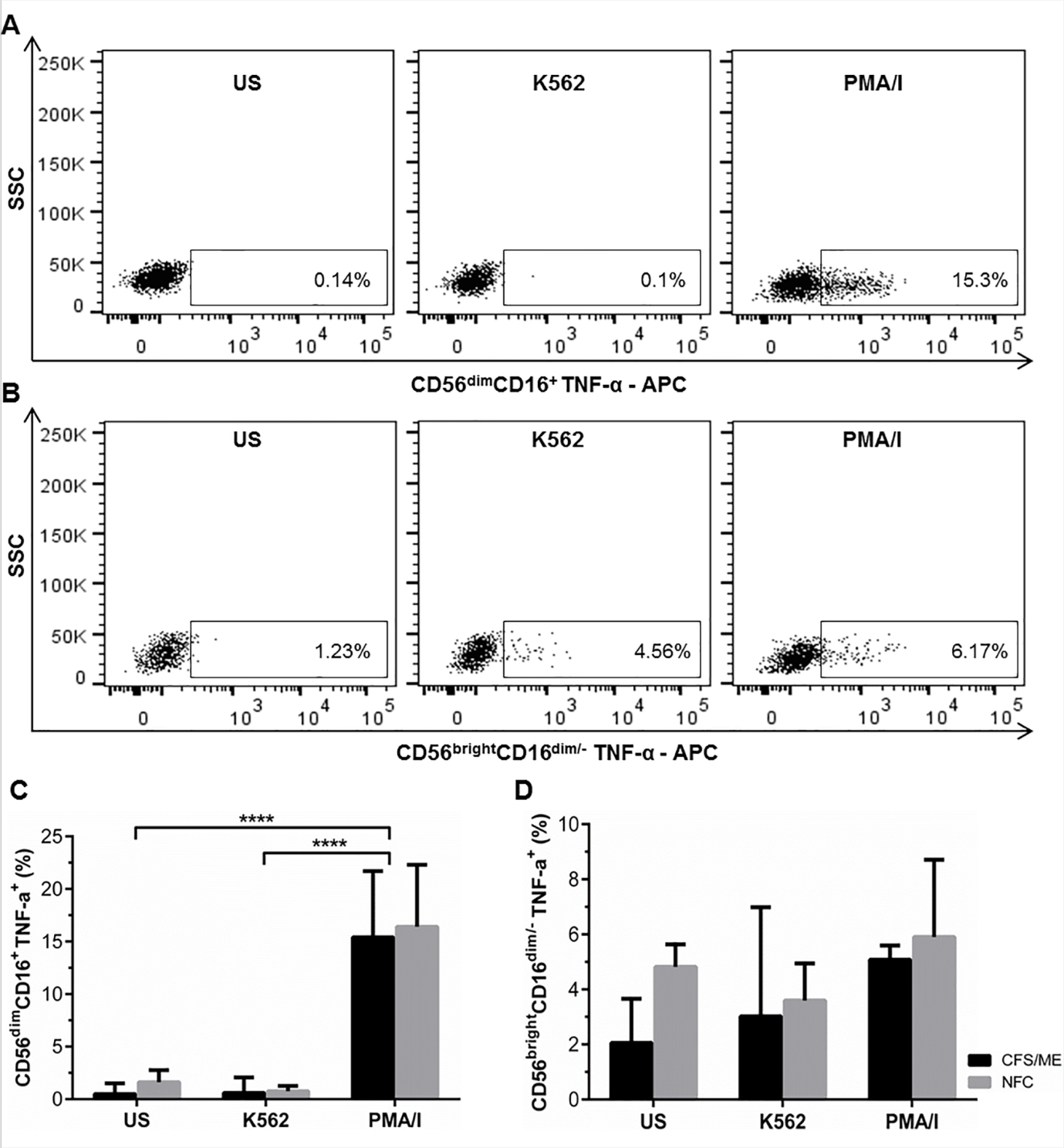


**Figure S17:** Flow cytometric analysis of GM-CSF production in CD56^dim^CD16^+^ (A) and CD56^bright^CD16^dim/-^ (B) NK cells. Production of GM-CSF in CD56^dim^CD16^+^ (C) and CD56^bright^CD16^dim/-^ (D) NK cells were not significantly different when compared between CFS/ME and NFC cohorts. Stimulation with PMA/I caused a significant increase in CD56^dim^CD16^+^ and CD56^bright^CD16^dim/-^ GM-CSF production in both CFS/ME and NFC compared to US and K562 incubated cells (****p<0.0001, ***p<0.001).


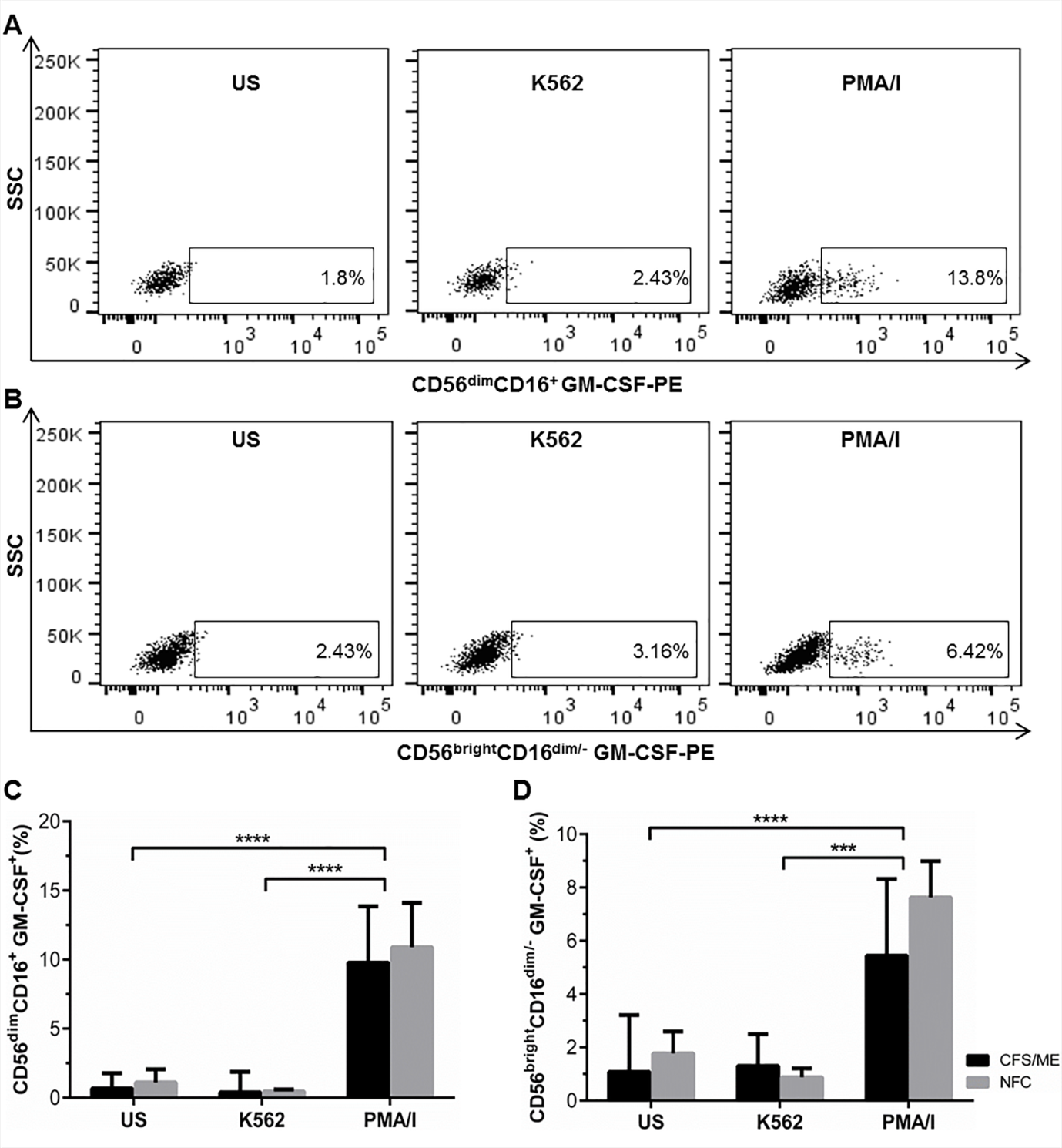

Supplement: Supplementary file 4 — 10.1186/s12967-016-0859-z NK cell cytokine results for CFS/ME patients and NFC. [file 12967_2016_859_MOESM4_ESM.docx]
